# Molecular basis of hERG potassium channel blockade by the class Ic antiarrhythmic flecainide

**DOI:** 10.1016/j.yjmcc.2015.06.021

**Published:** 2015-09

**Authors:** Dario Melgari, Yihong Zhang, Aziza El Harchi, Christopher E. Dempsey, Jules C. Hancox

**Affiliations:** a School of Physiology & Pharmacology, Medical Sciences Building, University Walk, Bristol BS8 1TD, UK; b School of Biochemistry, Medical Sciences Building, University Walk, Bristol BS8 1TD, UK

**Keywords:** Flecainide, hERG, QT interval, Repolarization, Torsades de Pointes

## Abstract

The class Ic antiarrhythmic drug flecainide inhibits *KCNH2*-encoded “hERG” potassium channels at clinically relevant concentrations. The aim of this study was to elucidate the underlying molecular basis of this action. Patch clamp recordings of hERG current (I_hERG_) were made from hERG expressing cells at 37 °C. Wild-type (WT) I_hERG_ was inhibited with an IC_50_ of 1.49 μM and this was not significantly altered by reversing the direction of K^+^ flux or raising external [K^+^]. The use of charged and uncharged flecainide analogues showed that the charged form of the drug accesses the channel from the cell interior to produce block. Promotion of WT I_hERG_ inactivation slowed recovery from inhibition, whilst the N588K and S631A attenuated-inactivation mutants exhibited IC_50_ values 4–5 fold that of WT I_hERG_. The use of pore-helix/selectivity filter (T623A, S624A V625A) and S6 helix (G648A, Y652A, F656A) mutations showed < 10-fold shifts in IC_50_ for all but V625A and F656A, which respectively exhibited IC_50_s 27-fold and 142-fold their WT controls. Docking simulations using a MthK-based homology model suggested an allosteric effect of V625A, since in low energy conformations flecainide lay too low in the pore to interact directly with that residue. On the other hand, the molecule could readily form π–π stacking interactions with aromatic residues and particularly with F656. We conclude that flecainide accesses the hERG channel from the cell interior on channel gating, binding low in the inner cavity, with the S6 F656 residue acting as a principal binding determinant.

## Highlights

• It is well known that hERG K^+^ channels regulate cardiac action potential repolarization.
• Molecular determinants of hERG block by the Class Ic antiarrhythmic flecainide were studied.
• Internal application of a charged flecainide analogue exerted block.
• The F656 but not Y652 S6 helical residue was crucial for flecainide binding.
• Direct interactions with pore helical residues are not necessary for flecainide binding.

## 1 Introduction

Flecainide is a class Ic antiarrhythmic drug that is used primarily in the treatment of supraventricular arrhythmias [1] although, in the absence of structural heart disease, it is sometimes used in the treatment of ventricular tachycardias that are resistant to other treatment. Guidelines from the American College of Cardiology, American Heart Association and European Society of Cardiology, recommend flecainide as a front-line treatment option for pharmacological cardioversion of atrial fibrillation (AF) and for the maintenance of sinus rhythm in AF patients (e.g. [2–4]). A 2011 review of 25 years of flecainide use supports the notion that, with appropriate patient selection, flecainide is safe and effective in AF management [5]. The importance of appropriate patient selection arises, to a significant extent, from the results of the CAST trial, which showed increased mortality with class Ic (flecainide and encainide) drug treatment of patients surviving myocardial infarction (MI) [6]. Recent evidence supports an additional clinical application of flecainide in the treatment of catecholaminergic polymorphic ventricular tachycardia (CPVT), particularly in cases where arrhythmias are not completely suppressed by β-blocker therapy (e.g. [7–10]).

Flecainide's class Ic action is attributable to the drug's open state dependent Na channel block and its Na channel association/dissociation kinetics [1,5,11]. The drug can prolong ventricular and atrial action potentials (APs), but shortens Purkinje fibre APs (reviewed in [5]). Therapeutic serum levels of flecainide lie between ~ 0.5 and 2.4 μM [12]. Flecainide inhibits L-type calcium current at concentrations that considerably exceed this range (IC_50_ of ~ 20 μM; [13]). Flecainide may also act as an open-channel blocker and gating modifier of the RyR2 ryanodine receptor [7,14]. It is also an effective inhibitor of cardiac K^+^ channels [15]. For example, flecainide discriminates between Kv4.x and 1.4 channels that contribute to transient outward K^+^ current (being more effective against the former subtype; [16]). It has also been shown to inhibit the rapid delayed rectifier K^+^ current, I_Kr_ [17–19] and recombinant channels encoded by *human Ether-à-go-go-Related Gene* (*hERG*; [20–24]). The agent's I_Kr_/hERG blockade occurs at concentrations that overlap the clinical concentration range [20,21,24] and, as noted by Aliot and colleagues, the drug's inhibitory effects on I_Na_ and I_Kr_ occur at lower concentrations than those on other channels [5]. They are therefore the ion channel effects that are likely to predominate during clinical use of the drug. Although rarely associated with *Torsades de Pointes* (TdP) arrhythmia or QT interval prolongation, some cases have been reported in which rate-corrected QT interval prolongation was not fully accounted for by widening of the QRS complex, and/or that exhibited a reverse-rate/bradycardia dependence that is consistent with a role for I_Kr_ inhibition (e.g. [25–28]).

Flecainide has been reported to exhibit characteristics of a comparatively rapidly acting, gated state dependent hERG channel blocker [20] and, in marked contrast to high affinity methanesulphonanilide inhibitors of hERG current (I_hERG_) [29,30], flecainide block of I_hERG_ develops rapidly on membrane depolarization [20]. Other class I antiarrhythmics that have been investigated in detail have shown a restricted set of binding residues and a relatively modest dependence of hERG channel blockade on inactivation gating [31–34]. In contrast, data on the underlying basis of flecainide inhibition of I_hERG_ are at present lacking. The present study was conducted to address this deficit, through investigation of the role of I_hERG_ inactivation and the roles of major pore helical and S6 helical residues in flecainide's inhibitory action.

## 2 Methods

### 2.1 Wild-type and mutant hERG channels

The mammalian cell line (Human Embryonic Kidney HEK293) stably expressing wild-type (WT) hERG channels was kindly donated by Prof Craig January [30]. The pore helix (T623A, S624A and V625A) and S6 helix (G648A and F656A) alanine mutants and the attenuated-inactivation mutants S631A, N588K and N588K/S631A were generated and used as described previously [33–36]. The HEK293 cell line stably expressing the hERG S6 helix mutant Y652A was used as described previously [35].

### 2.2 Maintenance of mammalian cells lines and cell transfection

HEK cells stably or transiently expressing hERG channel constructs were cultured as previously described [34,37]. The cells were plated in 40 mm petri dishes at least 48 h before transfection and incubated at 37 °C (5% CO_2_). Cells were transfected with Lipofectamine™ LTX (Invitrogen) following the manufacturer's instructions. The amount of transfected hERG construct DNA varied between 0.2 and 1.0 μg depending on the level of functional expression (assessed as current magnitudes) of each hERG channel construct. 0.5 to 1.0 μg of CD8 was co-transfected as a transfection-marker and successfully transfected cells were identified using Dynabeads® (Invitrogen). Cells were plated on small sterilized glass shards in 40 mm petri dishes and incubated at 37 °C (5% CO_2_) for at least 24 h before electrophysiological recording.

### 2.3 Electrophysiology

Glass shards containing plated cells were placed in a recording chamber mounted on an inverted microscope (Nikon Diaphot, USA). The cells were continuously superfused with a pre-warmed (37 °C) standard Tyrode's solution containing (in mM) the following: 140 NaCl, 4 KCl, 2.5 CaCl_2_, 1 MgCl_2_, 10 Glucose, 5 HEPES (titrated to pH 7.4 with NaOH). A modified “High K^+^” version of this solution (containing 94 mM KCl and 50 mM NaCl) was used to elicit recordable currents from T623A, G648A and F656A hERG channels mutants [34,37]. Glass patch-pipettes (Schott #8250 glass, A-M Systems Inc., USA) were pulled (Narishige, PP 830) and polished (Narishige, MF 83) to obtain a final resistance between 2 and 4 MΩ. Patch-pipettes were dialysed with an intracellular solution containing (in mM) the following: 130 KCl, 1 MgCl_2_, 5 EGTA, 5 MgATP, 10 HEPES (titrated to pH 7.2 with KOH). All recordings were made using an Axopatch 200B amplifier (Axon Instruments, now Molecular Devices) and a CV203BU head-stage. Pipette resistance compensation was between 70 and 80%. Data were acquired using a Digidata 1320 interface (Axon Instruments, now Molecular Devices). Data digitization rates were 10–25 kHz during all voltage protocols and an appropriate bandwidth of 2–10 kHz was set on the amplifier.

### 2.4 Data analysis and statistics

Data analysis was performed using Clampfit 10.3 (Axon Instruments, now Molecular Devices), Prism v4.03 and 5.03 and Excel 2013. Data are presented as the mean ± SEM or as mean with ± 95% Confidence Interval (CI). Equations used to fit particular data-sets are given in the online supplement. For statistical analysis, data were subject to the Kolmogorov–Smirnov normality test. Statistical comparisons were made using paired or unpaired two-tailed t tests, Wilcoxon matched pairs signed rank test, Mann–Whitney U test and one way (repeated measures, where indicated) or two way ANOVA, as appropriate. Details of the statistical test used to evaluate significance for results of particular experiments are given alongside “p” values in the Results text or in the relevant table or figure legend. p values less than 0.05 were taken as statistically significant.

### 2.5 Molecular modelling and docking

A homology model of the open configuration of the hERG channel pore region (pore helix, selectivity filter and S6 helices) based on the crystal structure of MthK channel [38] was used to perform docking simulations with flecainide using GOLD. Further details are provided in the online supplement.

## 3 Results

### 3.1 Effect of flecainide on WT hERG in different experimental conditions

**Fig. 1:**
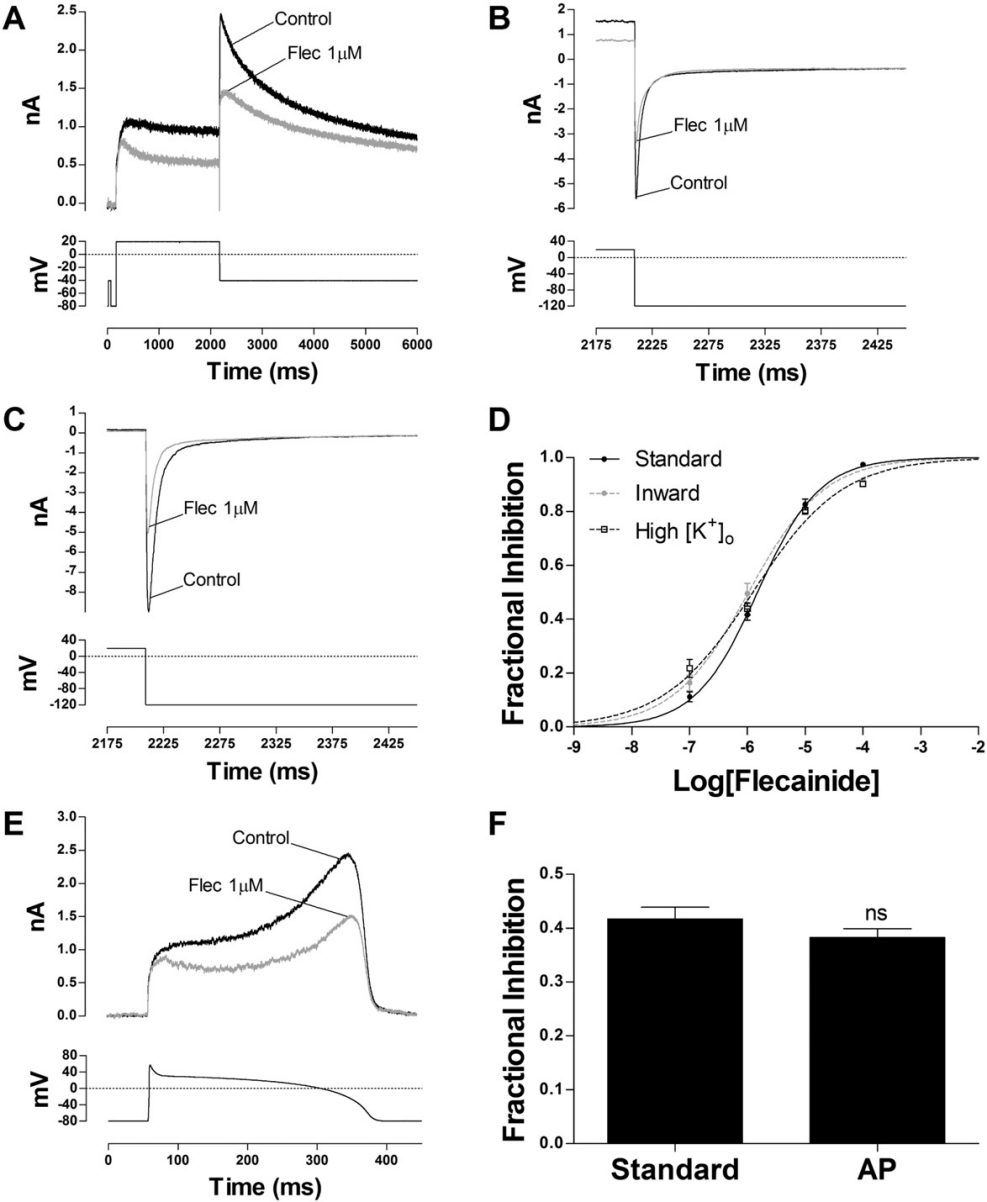
Effect of flecainide on WT I_hERG_. (A) Upper traces show WT I_hERG_ elicited by a standard protocol (lower trace) before (black) and during (grey) exposure to 1 μM flecainide. (B) Inward WT I_hERG_ (upper traces) measured at − 120 mV following an activating command to + 20 mV, before (black) and during (grey) exposure to 1 μM flecainide. The current traces are displayed to focus on the portion corresponding to repolarization from + 20 to − 120 mV. (C) Inward WT I_hERG_ measured at − 120 mV following an activating command to + 20 mV in high [K^+^]_o_ (94 mM) before (black) and during (grey) exposure to 1 μM flecainide. (D) Concentration–response relations for flecainide inhibition of WT I_hERG_ elicited under the different experimental conditions in A, B and C (n ≥ 5 at each concentration). IC_50_ and n_H_ values are given in the Results text. (E) Upper traces show WT I_hERG_ elicited by an AP voltage command before (black) and during (grey) exposure to 1 μM flecainide. (F) Comparison between the effect of 1 μM flecainide on WT I_hERG_ elicited by the standard protocol shown in ‘A’ (n = 21) and the AP voltage command (n = 6) (ns, p > 0.05 unpaired t-test vs. Standard).

Concentration-dependent block of WT I_hERG_ by flecainide was studied by the repeated application (start-to-start interval of 12 s) of the voltage protocol shown in the lower panel of Fig. 1A (see also [24,34,37]). Block of I_hERG_ was assessed by calculating the reduction in drug of the outward ‘tail’ current at − 40 mV (assessed as the difference in magnitude between peak tail current and instantaneous current activated by the brief (50 ms) depolarization from − 80 to − 40 mV [24,34,37]). Flecainide acted rapidly [20], and steady-state inhibition was attained within 3 min of drug application. Fig. 1A shows representative records of WT I_hERG_ in the absence (control) and in the presence of flecainide (1 μM). The fractional inhibition at each of four concentrations was calculated using Eq. (1) (supplementary information) and the mean values were plotted as shown in Fig. 1D and then fitted with a standard Hill equation (Eq. (2), supplementary information). This yielded a half-maximal inhibitory concentration (IC_50_) of 1.49 μM (CI: 1.27–1.74) with a Hill coefficient (n_H_) of 0.81 (CI: 0.68–0.93), in accord with previously observed values under similar experimental conditions [20,24]. Consistent with previous data [20], the inhibitory effect of flecainide on I_hERG_ exhibited voltage dependence (Fig. S1), with a concomitant leftward shift (~–7 mV) in voltage dependent activation. The low expression and kinetics of some mutant channels included in this study requires that I_hERG_ be studied as an inward current in high (94 mM) [K^+^]_o_ [34,35,37]. Consequently, flecainide block of WT I_hERG_ was also studied under comparable experimental conditions. Fig. 1B and C show the effect of 1 μM flecainide on inward WT I_hERG_ elicited at − 120 mV in normal Tyrode's and in High [K^+^]_o_ Tyrode's respectively; concentration response relations under these conditions are plotted in Fig. 1D. The fits to the experimental data gave an IC_50_ of 1.05 μM (CI: 0.82–1.38) with a n_H_ of 0.67 (CI: 0.57–0.77) for inward WT I_hERG_ in normal Tyrode's solution and an IC_50_ of 1.25 μM (CI: 0.97–1.60) with a nH of 0.56 (CI: 0.48–0.67) for inward WT I_hERG_ in High [K^+^]_o._. Thus flecainide inhibition of I_hERG_ was not sensitive to the direction/magnitude of K^+^ flux. Under action potential (AP) voltage clamp [37], peak I_hERG_ during AP repolarization (Fig. 1E) was inhibited by 38.3 ± 1.7% (n = 6), which is not significantly different from the fractional inhibition of the tail current (with the standard protocol) induced by the same drug concentration (41.7 ± 2.2% (n = 21); Fig. 1F, p > 0.05 unpaired t-test).

### 3.2 Effect of flecainide analogues on WT hERG channels

**Fig. 2:**
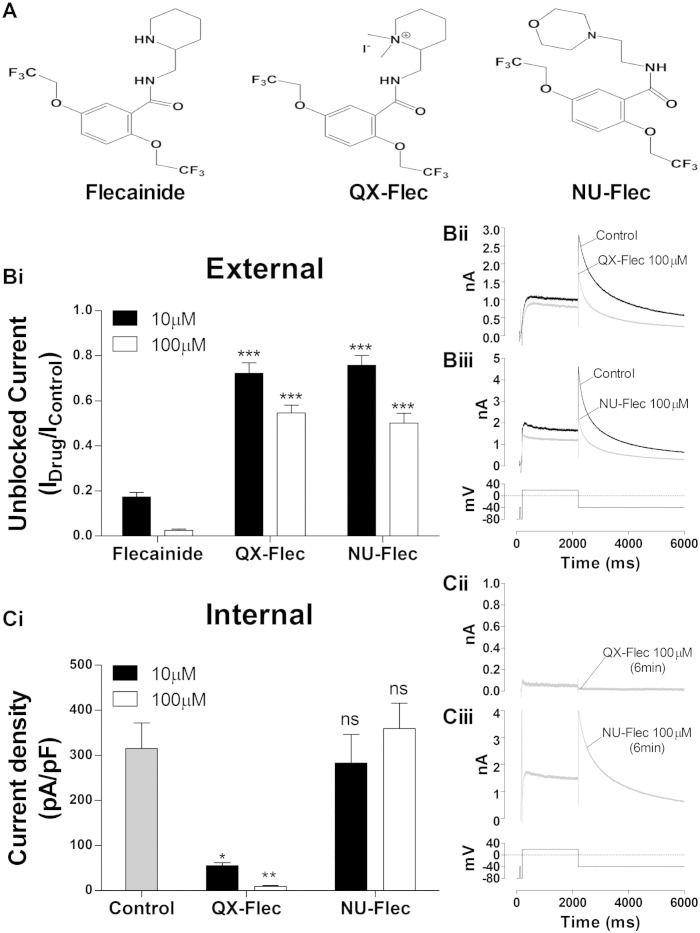
Effect of flecainide analogues on WT I_hERG_. (A) Chemical structure of flecainide (left, pKa 9.3), QX-Flec (centre) and NU-Flec (right, pKa 6.3) drawn with ChemBioDraw Ultra 14.0 (CambridgeSoft). (Bi) External application of two concentrations (10 μM, black and 100 μM, white) of flecainide (n = 10 and 6 respectively), QX-Flec (n = 9 and 10 respectively) and NU-Flec (n = 8 and 10 respectively); data are shown as Unblocked Current remaining after drug exposure (plotted as I_Drug_/I_Control_) (***p < 0.001 one-way ANOVA with Bonferroni post hoc). (Bii, Biii) Sample traces of WT I_hERG_ before (black) and during (grey) the external application of (Bii) QX-Flec 100 μM and (Biii) NU-Flec 100 μM. (Ci) Current density for I_hERG_ (pA/pF) with internal application (via the patch pipette) of two concentrations (10 μM, black and 100 μM, white) of QX-Flec (n = 7 and 5 respectively) and NU-Flec (n = 6 and 12 respectively) compared with I_hERG_ density with normal (drug-free) pipette solution (grey, n = 10. i.e. normal pipette solution; *p < 0.05, **p < 0.01 and ns p > 0.05, one-way ANOVA with Bonferroni post hoc vs. control). (Cii, Ciii) Sample traces of WT I_hERG_ (grey) recorded at 6 min of internal exposure (from gaining whole cell access) to (Cii) QX-Flec 100 μM and (Ciii) NU-Flec 100 μM.

Membrane “sidedness” of flecainide action was explored using two flecainide analogues used in prior investigation of the drug's block of sodium channels (Fig. 2A — a fully charged flecainide analogue QX-Flec and the ~ 90% neutral analogue NU-Flec (pKa 6.3) [39]). We tested the effect of two concentrations (10 and 100 μM) of each analogue applied from the cell exterior (superfusate) and interior (the latter through the patch-pipette). Fig. 2Bi summarizes the results from the external application of the analogues compared to the parent compound. Sample traces of I_hERG_ elicited by a standard voltage protocol in control and in the presence of 100 μM of QX-Flec and Nu-Flec are shown in Fig. 2Bii and Biii respectively. Each analogue was superfused over the cell under study for 6–8 min and both exhibited a reduction in potency compared to flecainide. For 10 and 100 μM QX-Flec, the fraction of unblocked I_hERG_ was 72.2 ± 4.6% (n = 9) and 54.6 ± 3.5% (n = 10) respectively, whilst 10 and 100 μM NU-Flec left an unblocked current fraction of 75.8 ± 4.4% (n = 8) and 50.2 ± 4.3% (n = 10) respectively (p < 0.001, one-way ANOVA with Bonferroni post hoc test). Pipette application of two different concentrations of QX-Flec and NU-Flec from the patch-pipette was determined. As soon as the whole-cell configuration was achieved the drug molecules were free to diffuse inside the cytoplasm, precluding cells acting as their own control. Instead, time matched (~ 6 min) measurements of I_hERG_ density (with 10 or 100 μM QX-Flec or NU-Flec; Fig. 2Ci) were compared with measurements from cells dialysed with drug-free standard intracellular solution. Fig. 2Cii and Ciii show I_hERG_ traces after 6 min of internal perfusion with 100 μM QX-Flec and NU-Flec respectively. Mean values are reported in Fig. 2Ci. When dialyzed with 10 or 100 μM QX-Flec the whole-cell current density was 55.3 ± 5.9 pA/pF (n = 7) and 9.3 ± 1.9 pA/pF (n = 5) respectively, and both values are significantly different from control (315.3 ± 56.7 pA/pF (n = 10), p < 0.05 and p < 0.01 respectively, one-way ANOVA with Bonferroni post hoc). Conversely, there was no significant difference in I_hERG_ density between control cells and cells dialyzed with 10 or 100 μM NU-Flec (283.2 ± 63.4 pA/pF (n = 6) and 359.8 ± 56.1 pA/pF (n = 12) respectively, p > 0.05 one-way ANOVA with Bonferroni post hoc). The results of these experiments are consistent with prior data for SCN5A sodium channels, for which both analogues were less effective when applied from the cell exterior, whilst QX-Flec was particularly effective when applied directly to the cell interior [39].

### 3.3 Relationship between flecainide block and hERG channel inactivation

**Fig. 3:**
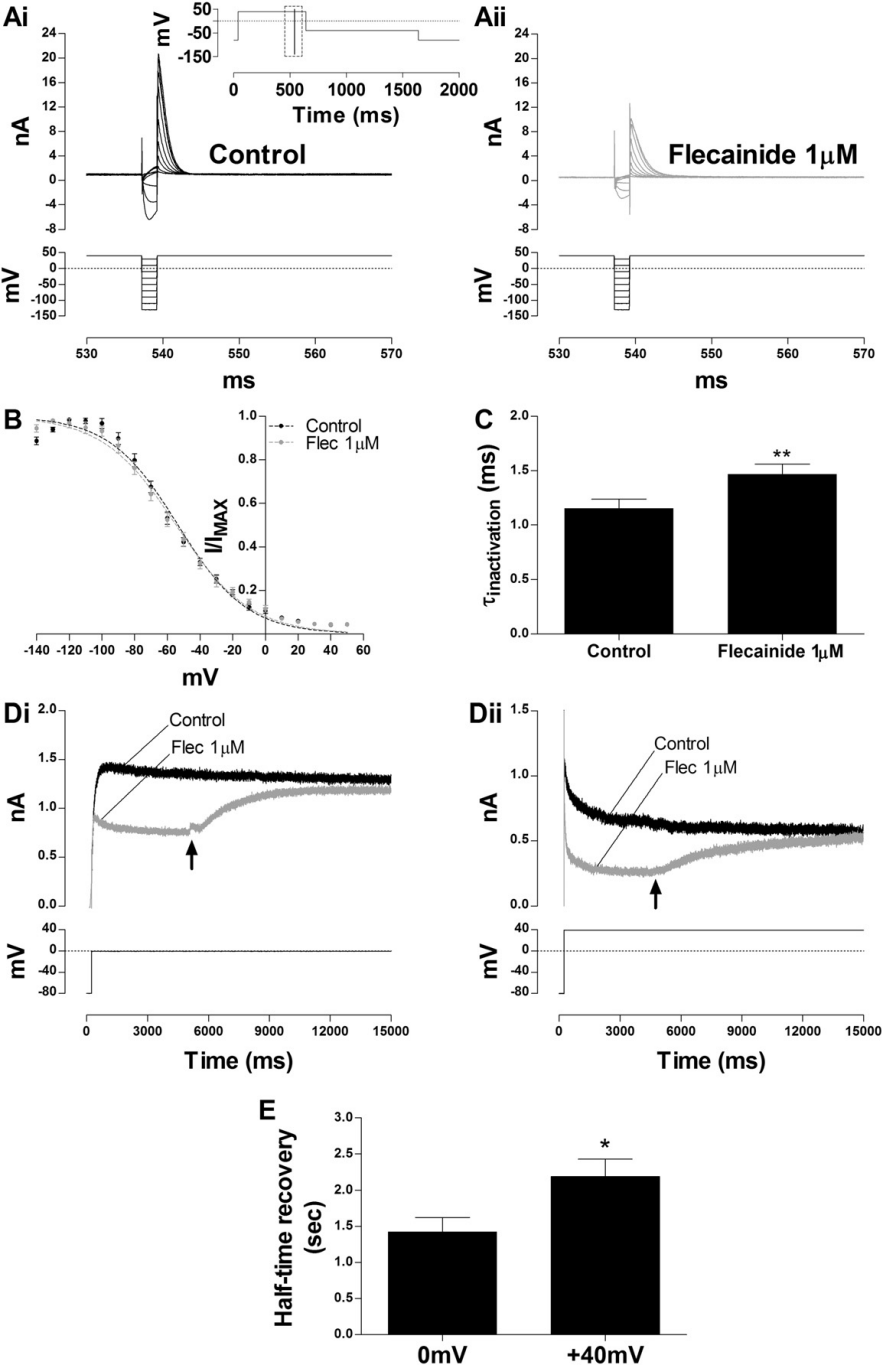
WT I_hERG_ inactivation and flecainide. (A) Upper traces show WT I_hERG_ elicited by the “availability protocol” (from a holding potential of − 80 mV the membrane was depolarized to + 40 mV (500 ms) and then briefly (2 ms) repolarized to a test potential ranging from − 140 mV to + 50 mV before returning to + 40 mV. Full protocol shown in the inset; the traces focus on the boxed area from the full protocol) in control (Ai) and in the presence of 1 μM flecainide (Aii). (B) Voltage dependence of inactivation for WT I_hERG_ in control (black circles) and after the application of 1 μM flecainide (grey circles) (n = 6, ns p > 0.05 Wilcoxon matched-pairs signed rank test). V_0.5_ and k values are given in the Results text. (C) Time constants of inactivation of WT I_hERG_ at + 40 mV after a hyperpolarizing step to − 120 mV in control and in the presence of 1 μM flecainide (**p < 0.01, paired t-test, n = 6). (D) Representative traces of WT I_hERG_ elicited by a long (15 s) depolarizing step to 0 mV (Di) and + 40 mV (Dii) in control (black) and in the presence of 1 μM flecainide (grey). Black arrows indicate the time at which superfusate was switched from drug to control, in order to wash out flecainide. (E) Half-time recovery of the current recovery from flecainide block during a long depolarizing pulse at 0 mV and + 40 mV (*p < 0.05, unpaired t-test. n = 5 for both).

I_hERG_ inhibition by a number of cardiac and non-cardiac drugs is influenced significantly by channel inactivation (e.g. [31,33,40,41]), although class I antiarrhythmic drugs examined to date appear not to depend strongly on inactivation to block the channel [31,33,34]. Fig. 3A and B show the effect of flecainide on I_hERG_ availability, studied with the three-step protocol reported in the inset of Fig. 3Ai [36,42]. The resurgent transient current elicited at the beginning of the third step at + 40 mV was analysed as previously described [36,42]. Fig. 3Ai and Aii show sample traces of resurgent I_hERG_ in control and in the presence of 1 μM flecainide respectively (for clarity currents at selected voltages are shown). Mean values of normalized current were plotted against the corresponding test voltage during the 2 ms step (Fig. 3B) and fitted with a Boltzmann function (Eq. (3), supplement). This yielded a V_0.5_ of inactivation of − 53.4 ± 2.3 mV with a Slope factor of 20.9 ± 0.5 (n = 6) in the control and a V_0.5_ of inactivation of − 54.6 ± 2.9 mV with a Slope of 22.1 ± 0.9 (n = 6) in the presence of 1 μM flecainide (p > 0.05 for both parameters versus control; Wilcoxon matched-pairs signed rank test). Thus, flecainide did not significantly alter the voltage dependence of hERG channel inactivation. The effect of flecainide on the time-course of I_hERG_ inactivation was assessed by fitting the decay of the resurgent current elicited at the beginning of the third step after a hyperpolarizing step to − 120 mV with a monoexponential function (Eq. (4), supplement). The results are summarized in Fig. 3C. Flecainide slightly but significantly increased the time constant of inactivation at − 120 mV from 1.15 ± 0.09 ms to 1.47 ± 0.09 ms (n = 6, p < 0.01 paired t-test). In further experiments, the extent to which C-type inactivation might stabilize the binding of flecainide to hERG channels was investigated by washing the drug off during sustained depolarization. To do so we applied a long (15 s) depolarizing step to 0 or + 40 mV in control and in the presence of 1 μM flecainide (Fig. 3D). When the steady-state of block was reached the perfusion system was quickly switched back to control during the depolarizing pulse. The half-time of recovery (t_half_ recovery) was calculated. At 0 mV the t_half_ recovery was 1.42 ± 0.20 s (n = 5) and at + 40 mV it was 2.19 ± 0.24 s (n = 5) (Fig. 3E; p < 0.05, unpaired t-test). The slower recovery of macroscopic I_hERG_ at the more depolarized potential is suggestive that the drug is more tightly bound to hERG channels in the inactivated state.

**Fig. 4:**
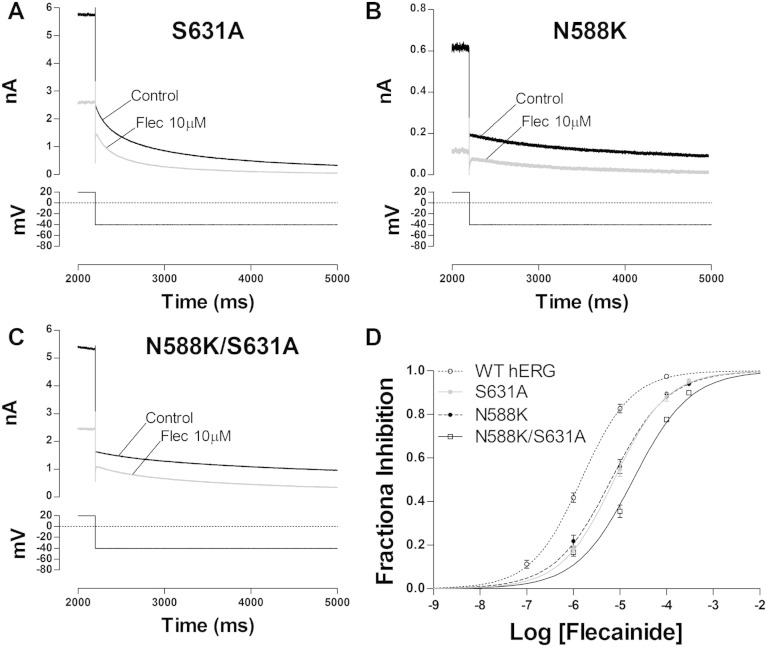
Flecainide block of I_hERG_ carried by attenuated-inactivation mutants. (A) Upper traces show S631A I_hERG_ elicited by a standard protocol (expanded portion shown as lower trace; protocol identical to that used to study WT I_hERG_ in Fig. 1A) in control (black) and in the presence of 10 μM flecainide (grey). (B) Upper traces show N588K I_hERG_ elicited by same protocol as ‘A’ in control (black) and in the presence of 10 μM flecainide (grey). (C) Upper traces show N588K/S631A I_hERG_ elicited same protocol as ‘A’ in control (black) and in the presence of 10 μM flecainide (grey). (D) Concentration response relations for the effect of flecainide on the three attenuated-inactivation hERG mutants: S631A (grey circles and grey dashed line), N588K (black circles, black dashed line) and N588K/S631A (white square, solid black line) compared with WT relation, replotted from Fig. 1D (white circles, dotted black line) (n ≥ 5 for each concentration of each curve).

The inactivation-dependence of flecainide block of hERG was studied further using mutants with impaired inactivation (Fig. 4). Different flecainide concentrations were tested on the S631A and N588K hERG mutations which share a similar rightward shift in the voltage dependence of inactivation [33], but which are located in different regions of the channel (the pore and S5-pore linker respectively). Effects were also studied of the N588K/S631A double mutant which is known to have a more severe impairment of channel inactivation than either single mutant [33]. Fig. 4A–C shows expanded current traces of these three mutants elicited by a standard protocol in control and in the presence of 10 μM flecainide. Experimental data were plotted and analysed for WT channels, with concentration–response relations shown in Fig. 4D. S631A and N588K showed similar sensitivities to flecainide, with an IC_50_ of 7.49 μM (CI: 6.33–8.87) and 6.50 μM (CI: 5.37–7.88) and n_H_ of 0.77 (CI: 0.68–0.85) and 0.72 (CI: 0.63–0.81) for S631A and N588K, respectively. This corresponds to a ~ 5-fold increase in the IC_50_ value for S631A and a ~ 4.4-fold increase for N588K compared with WT. The N588K/S631A double mutant showed a further shift in the dose–response relationship with an IC_50_ of 19.2 μM (CI: 15.3–23.8) with a n_H_ of 0.72 (CI: 0.62–0.82) which corresponds to a ~ 13-fold decrease in potency compared to WT.

### 3.4 Effect of mutations in the hERG pore helix on flecainide potency

**Fig. 5:**
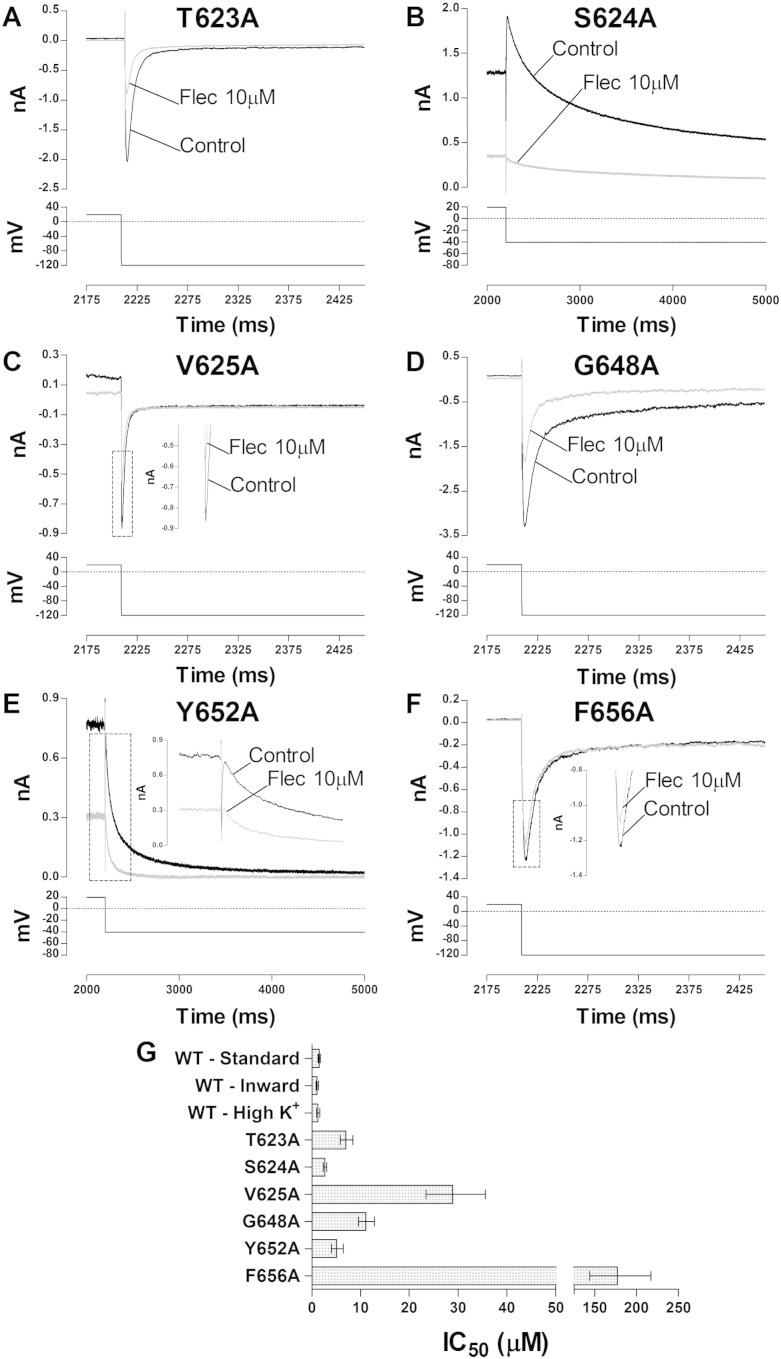
Effect of pore-helix and S6 helix mutations on flecainide inhibition of I_hERG_. (A–C) Upper traces show I_hERG_ elicited by protocols shown as lower traces in control and 10 μM flecainide for the T623A (A), S624A (B) and V625A (C) pore-helical mutants. (D–F) Upper traces show I_hERG_ elicited by protocols shown as lower traces in control and 10 μM flecainide for the G648A (D), Y652A (E) and F656A (F) S6 helix mutants. (G) IC_50_ for flecainide inhibition of the alanine mutants shown in A–F, together with those for their WT I_hERG_ comparators (WT “Standard” — for S624A and Y652A; WT “inward” for V625A; WT “High K^+^” for T623A, G648A and F656A). Plotted error bars represent 95% confidence intervals. Each IC_50_ was derived from a concentration–response relationship derived from at least four different drug concentrations, n ≥ 5 for each concentration on each curve.

A cluster of three residues (T623, S624 and V625) located in the lower portion of pore helix-selectivity filter region has been reported to be involved in the binding to hERG of different drugs (e.g. [43–45]). In order to determine the roles of these residues in flecainide block of I_hERG_ we studied alanine mutants of each of T623, S624 and V625. The T623A mutant was tested in High [K^+^]_o_ and the current was elicited by a hyperpolarizing step to − 120 mV in order to obtain an appreciable inward current transient. The S624A mutant was studied in normal Tyrode's solution and the current was elicited by a standard voltage protocol. The V625A mutant was also tested in normal Tyrode's solution but the current was elicited by the same hyperpolarizing protocol used for T623A [34,37]. Sample traces of T623A, S624A and V625A currents in control and in the presence of 10 μM flecainide are shown in Fig. 5A, B and C respectively. Different flecainide concentrations were tested and a full concentration–response relationship was constructed for each mutant. The derived IC_50_s are reported in Fig. 5G and Table 1 together with the values from WT hERG under matching experimental conditions (see Fig. 1A–D). The calculated IC_50_ for T623A was ~ 5.6-fold that for its WT control. For S624A the IC_50_ was ~ 1.8-fold its WT control. The IC_50_ for V625A was ~ 27-fold its WT control.

### 3.5 Effect of S6 helix mutations on flecainide potency

To investigate potential binding determinants in the pore region we studied alanine mutants of three residues (G648, Y652 and F656) located within the channel inner cavity on the S6 helix. G648 is a highly conserved residue involved in the binding of high affinity methanesulphonanilide agents [43,44], whilst the aromatic Y652 and F656 residues are of fundamental interest as interactions with at least one of the two have been found to be important for the majority of drugs tested [43–46]. G648A and F656A were studied under the same experimental condition as T623A, whilst Y652A was tested in normal Tyrode and the current was elicited by a standard voltage protocol. Sample traces for G648A, Y652A and F656A I_hERG_ in control and in the presence of 10 μM flecainide are shown in Fig. 5D, E and F, respectively. Different concentrations were tested and the IC_50_ values from the resulting concentration response relationships are plotted in Fig. 5G for comparison with their respective WT controls, with mean IC_50_ and n_H_ values given in Table 1. The derived IC_50_ for G648A was ~ 9-fold its WT control, that for Y652A was 3.4-fold its WT control and that for F656A was 142-fold its WT control, with an n_H_ of 0.55 (CI: 0.47–0.63). These results support a particularly strong role for the F656 residue in flecainide binding. This was confirmed in further experiments using pipette application of the charged analogue QX-Flec, which produced little inhibition of F656A hERG (see Fig. S2).

### 3.6 Docking simulation of flecainide with a WT hERG model

**Fig. 6:**
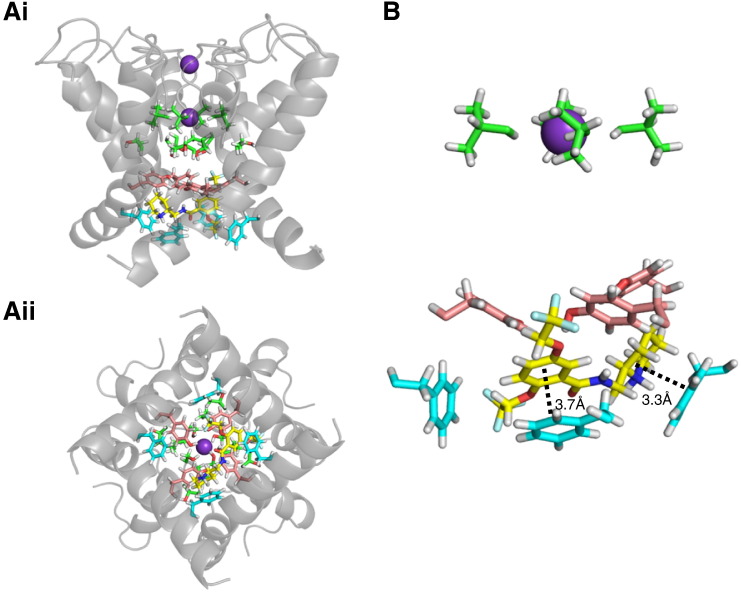
*In silico* docking simulations of flecainide to hERG. (A) Lateral (Ai) and intracellular views (Aii) of a representative low energy pose of flecainide (yellow sticks) docked to an MthK-based open-state hERG channel homology model. Pore region domains are shown as grey ribbons. Green sticks represent residues T623, S624 and V625, whilst Y652 and F656 are shown as pink and blue sticks respectively. K^+^ ions in the selectivity filter are shown as purple spheres. (B) Representative low energy GOLD pose for flecainide (yellow sticks) docked to hERG pore cavity residues. Residues V625, Y652 and F656 are shown as green, pink and blue sticks, respectively. For clarity only three F656 side chains and three Y652 side chains are shown. The interactions included a π–π interaction (3.7 Å) between the aromatic ring of the benzamide moiety of flecainide and F656, and a second π–π interaction (3.3 Å) between the piperidine ring and an adjacent F656 (black dotted lines).

Recent studies have proposed different models of flecainide binding to Kv2.1 and Kv1.5 channels [47,48]. In those models flecainide was proposed to interact with potassium sites in the central cavity and with residues located at the subunit interface between S6 and P-helices. Here we tested flecainide on a MthK-based hERG channel model [38] using the docking program GOLD. Several different low energy conformations were obtained that broadly conformed with the experimental observations; a sample pose is shown in Fig. 6. The molecule tended to adopt an extended conformation, and in most of the low energy poses flecainide docked low in the pore cavity lying between the two aromatic residues F656 and Y652. In this position the piperidine ring and the benzamide moiety of flecainide formed stacking π–π interaction with the surrounding cluster of aromatic rings, in particular with F656 (Fig. 6B). This is consistent with the experimental data in showing a strong dependence of flecainide binding on the presence of the F656 aromatic side chain. This simulation also suggests an allosteric role of V625 residue in the selectivity filter region, since the drug molecule lies too far away to develop a direct interaction with V625. Similar results were obtained in a second series of docking runs using the program Flexidock which better accounts for electrostatic contributions to binding [38] (see Supplement). For other drug molecules Flexidock tends to locate the positively charged portion of the drug closer to the selectivity filter and in or near the binding site for K^+^ ions. However, in our analyses with flecainide most of the low energy conformations had flecainide docked to the inner mouth of the pore interacting mainly with F656 aromatic rings (see Fig. S3).

## 4 Discussion

Flecainide was originally shown to inhibit feline ventricular E-4031 sensitive delayed rectifier current with an IC_50_ of 2.1 μM [17,18]. At high concentrations (10 and 30 μM) it was found to inhibit guinea-pig ventricular I_Kr_ with selectivity over the slow delayed rectifier current, I_Ks_ [19]. Subsequently reported IC_50_ values for flecainide inhibition of WT I_hERG_ from mammalian expression systems range from 0.74 to 3.91 μM [20,21,24]. The observed potency of flecainide against WT I_hERG_ in the present study falls within this range and is consistent with the potential for direct I_Kr_/I_hERG_ inhibition at clinical concentrations [12]. In addition to exerting an acute inhibitory effect on I_hERG_, some drugs are also able to impair hERG trafficking [49–51]. Indeed, in one study, 40% (20 of 50) of drugs producing a direct inhibitory effect on I_hERG_ also produced trafficking defects [50]. The focus of the present study was to establish molecular determinants of the acute I_hERG_ inhibitory effects of flecainide; the drug's effects on hERG channel trafficking were not investigated and remain to be established.

### 4.1 Gating dependence of flecainide block and intracellular access to the drug binding site

Flecainide has been reported to exhibit both (rapid) time and voltage-dependent inhibition of I_hERG_ [20] and the present study (Fig. S1) affirms voltage-dependence of the drug's action. The role of inactivation gating in the drug's action has not hitherto systematically been investigated. The lack of leftward shifted WT I_hERG_ inactivation seen here with flecainide suggests that the drug does not act strongly to stabilize the inactivated state, although the time-course of inactivation was modestly slowed by the drug (Fig. 3). On the other hand, the slower washout of flecainide during sustained depolarization to + 40 mV compared to 0 mV, which promoted I_hERG_ inactivation (Fig. 3D, E), suggests that inactivation plays a role in stabilizing the drug within the channel. Our experiments with the S631A and N588K single and double mutations (Fig. 4), yielded an increased IC_50_ for inhibition of channels with impaired inactivation, which further supports this notion. The ~ 4–5 fold shift in IC_50_ with the N588K and S631A mutations and ~ 13-fold shift for the double mutant compare with an IC_50_ for I_hERG_ inhibition by the methanesulphonanilide E-4031 that was increased 11–12 fold by the two single mutants and ~ 36-fold by the N588K/S631A double mutant [33]. Table S1 shows further comparisons of the effects of inactivation mutations on the action of flecainide with comparable values for other class I and III agents. Considered in this context, flecainide inhibition of I_hERG_ shows greater dependence on inactivation than other class I agents studied to date, but less dependence than that observed for methanesulphonanilides.

In a prior study from our laboratory, intracellular dialysis via the patch pipette of flecainide produced less inhibition of I_hERG_ than did extracellular application of the same drug concentration [24]. Additionally, extracellular flecainide block of I_hERG_ was also not sensitive to acidification of the pipette solution (although it was to extracellular acidosis). These observations led to a suggestion that flecainide may not access its binding site on the hERG channel from the cell interior [24]. These prior observations are consistent with results on flecainide block of native cardiac I_Na_ and *SCN5A*-encoded recombinant sodium channels, as — despite acting as an open channel blocker — intracellular flecainide (again applied via the patch pipette) had been reported to be relatively ineffective at inhibiting I_Na_ [39,52]. In experiments using QX-Flec and NU-Flec, Liu and colleagues were able to resolve the membrane ‘sidedness’ of flecainide block and the role of drug ionization in Na channel block [39]. They found that permanently charged flecainide was effective only when applied to the cell interior [39]. Flecainide has a pKa value of 9.3, meaning that the majority, but not all of the parent drug would be expected to be ionized at normal extracellular and intracellular pH values. Liu et al proposed that the apparent ineffectiveness of parent flecainide when applied internally could be explained by a scheme in which the small neutral component of drug leaves the cell by crossing the cell membrane and is then rapidly diluted and washed away by external superfusate, with intracellular re-equilibration/partition between charged and uncharged drug then followed by further uncharged drug egress, leading to dramatic attenuation of action of the pipette-applied flecainide (see [39]). This interpretation may also explain prior observations in respect of pipette applied flecainide on I_hERG_ [24]. In our experiments with QX-Flec and NU-Flex on hERG, the former but not latter compound was an effective inhibitor when applied via the patch pipette. The modest component of I_hERG_ inhibition seen when QX-Flec was externally applied is consistent with some passage across the membrane of the quaternary analogue (cf [53]). Thus, the data in the present study are consistent with a scheme in which externally applied flecainide crosses the cell membrane and once inside the cell interior the charged fraction of drug enters the channel on activation gating to exert its inhibitory effect. Independent support for the idea that flecainide acts to inhibit hERG from the cell interior comes from the observation that the drug's action is potentiated in cells transfected with the drug uptake transporter OCTN1 [22].

### 4.2 Binding determinants within the hERG inner cavity

High affinity pharmacological inhibition of hERG channels is characterized by drug interactions with a binding site in the inner cavity involving multiple actions with pore-helical and S6 residues (e.g. [43,44,53–55]). For the methanesulphonanilide agent MK499, the Y652A mutation increased the I_hERG_ IC_50_ 94-fold, whilst F656A increased it 650 fold. G648A resulted in an IC_50_ 55-fold that of WT I_hERG_ whilst for T623A, and V625A the corresponding increases were 5 fold and 50 fold, respectively [43]. The related agents E-4031 and dofetilide have similar binding determinants [44]. In contrast, high affinity block by both cisapride and terfenadine has been reported to involve both S6 aromatic residues and T623 and S624 pore-helical residues, but not V625, G648 and V659 residues, indicating that different molecules producing high affinity block can adopt distinct binding modes within the channel's inner cavity [43,54]. By comparison with these drugs, flecainide has a more restricted set of binding determinants. In the case of the cardiac Na channel flecainide shares consensus tyrosine (Y) and phenylalanine (F) binding residues (F1760 and Y1767) on the SCN5A-encoded protein to those of lignocaine [39], whilst for Kv1.5 mutating one of the identified binding determinants, V505, to phenylalanine increased blocking potency 9-fold [56]. For flecainide, our data indicate that F656 but not Y652 acts as a key determinant of I_hERG_ inhibition, the crucial role for F656 being further demonstrated by the loss of a significant inhibitory effect of QX-Flec with the F656A mutation. For the class Ia antiarrhythmic quinidine the F656V mutation increased the I_hERG_ IC_50_ by ~ 27 fold [31], whilst F656A and Y652A mutations increased I_hERG_ IC_50_ for disopyramide respectively by ~ 55 and ~ 51 fold respectively [34], and so flecainide appears to be more sensitive to mutation of F656 than is either of these drugs. In prior experiments using *Xenopus* oocyte expression, the inhibitory action of the class Ia antiarrhythmic drug ajmaline on I_hERG_ was greatly impaired by both the Y652A and F656A mutations, as a profound blocking concentration (for WT I_hERG_; 300 μM) was found to be ineffective against both mutants, from which it is possible to conclude that the Y652 residue plays a more prominent role in the binding of ajmaline than of flecainide [57]. However, as full concentration–response data are not available for ajmaline and these mutants [57], quantitative comparison between ajmaline and flecainide of the relative importance of residue F656 for binding is not possible. To our knowledge, the only other class Ic drug for which hERG binding determinants have been investigated is propafenone [32]. Although structurally distinct from flecainide, propafenone shares with flecainide a relative lack of dependence of hERG block on interactions with Y652 (IC_50_ 6.3-fold that of WT I_hERG_) and a marked dependence on F656 (IC_50_ ~ 95-fold and ~ 56-fold the values for WT I_hERG_ in low and high [K^+^]_o_ respectively) [32]. Propafenone showed relatively little sensitivity to T623A, S624A and V625A mutations [32], whilst disopyramide has also been shown to be little affected by T623A and S624A, with only a small effect of V625A [34]. Although the effect of the V625A mutation on flecainide's I_hERG_ inhibitory action in vitro was marked, the *in silico* docking in the present study supports an indirect role for this residue as in low energy binding conformations direct interaction with V625 did not occur. Binding of flecainide relatively low in the pore cavity is further supported by the lack of effect of altering the direction of K^+^ flux (i.e. lack of a “knock off” effect), which suggests that flecainide is unlikely to lie near the K^+^ ion conduction pathway. The V625A mutation profoundly attenuates I_hERG_ inactivation [58]. Attenuated inactivation has been proposed to influence drug blocking potency by altering the positioning of S6 aromatic binding residues relative to the inner cavity [59], and this may account for the allosteric effect of V625A on flecainide binding. Support for such an explanation comes from the marked effect of the N588K/S631A double mutant, which exhibits little inactivation [33], on flecainide block, as neither N588 nor S631 lies near the binding site for flecainide.

### 4.3 Clinical relevance and implications

As commented in the Introduction text and highlighted by others [5], flecainide's inhibitory effects on I_Na_ and I_Kr_ occur at lower concentrations than those on other channels; they are therefore the ion channel effects that are likely to predominate during clinical use of the drug. Until recently, the drug's effectiveness against CPVT (e.g. [7–10]) has been considered to be a direct consequence of inhibition of RyR2 ryanodine receptors (e.g. [7,60]), which in intact cardiomyocytes would require cell entry of the drug to access RyR2s in the sarcoplasmic reticulum membrane. However, a very recent study has suggested that flecainide does not inhibit luminal-to-cytosolic cation flux through RyR2 and that the drug's beneficial actions in CPVT are secondary to Na^+^-dependent modulation of intracellular Ca^2 +^ ion handling [61]. The drug's inhibitory action on Na^+^ channels may also reduce oxidative stress at high atrial rates through reducing Na^+^ and, thereby, Ca^2 +^ accumulation [5]. Taken together, prior work on Na^+^ channels using flecainide analogues [39] and the results of the present study indicate that the drug's actions on its principal ion channel targets in the sarcolemmal membrane rely on charged drug access to the target channels from the cell interior.

Although flecainide is recommended as a front-line treatment option for both pharmacological cardioversion and maintenance of sinus rhythm in AF patients [2–4], data from the 2005 Euro Heart Survey on AF suggested that the actual clinical use of the drug is not high (with ~ 17% and 13% of paroxysmal and persistent AF patients receiving class Ic drug treatment) [5,62]. However, although the results of the CAST trial indicate clearly that flecainide should be avoided in patients with structural heart disease, a review of 25 years of clinical flecainide use suggests that the drug is a safe and effective treatment option for AF in younger patients without concomitant structural heart disease [5]. For example, one systematic review of randomized controlled trials found the incidence of ventricular arrhythmias in AF patients given flecainide to be 3% or less [63]. A meta-analysis of 122 studies of patients receiving flecainide for supraventricular tachycardias, without obvious signs of ventricular damage, found flecainide to be associated with a lower incidence of proarrhythmic events than control (2.7 vs. 4.8%) [64]. Clearly, though, flecainide's ability to inhibit I_Kr_/I_hERG_ at clinically relevant concentrations makes it important to exercise appropriate caution in either very carefully monitoring or avoiding the drug's administration to patients with other risk factors for acquired LQTS, such as pre-existing QT prolongation, co-administration with other I_Kr_/I_hERG_ blocking drugs, or hypokalemia.

## 5 Conclusions

Flecainide acts as a gated state inhibitor of I_hERG_ at clinically relevant concentrations. Our findings are consistent with a scheme in which flecainide crosses the cell membrane and accesses the channel on activation gating. Our data with QX-Flec and NU-Flec support the notion of preferential interactions between the charged form of the drug and a binding site within the hERG inner cavity. Flecainide exhibits a distinct profile of responses to mutations of amino-acid residues that form the canonical binding site for high affinity inhibitors. It binds low in the inner cavity and interacts strongly with F656. The effect of inactivation gating on flecainide block is likely to result from stabilization of drug binding by optimising drug interactions with F656 aromatic side chains.

## Disclosures

None.

## Figures and Tables

**Table 1:** Effect of pore helix and S6 mutations on I_hERG_ inhibition by flecainide. The table shows the effects of mutations in the pore-helix/selectivity filter region (T623A, S624A, V625A) and S6 helix (G648, Y652A, F656A) on I_hERG_ block by flecainide. WT-1, WT-2, WT-3 refer to the 3 conditions under which WT I_hERG_ IC_50_ values were obtained. For all channel variants, the voltage-step shows the membrane potential at which I_hERG_ tails were measured and the corresponding external potassium ([K^+^]_o_) values are given. For each channel/recording condition the mean IC_50_ and n_H_ values are given and for each mutant, the fold change in IC_50_ relative to its corresponding WT control is also given (IC_50_ mutant/IC_50_ of WT-control).

Channel	Voltage step mV	K^+^ mM	IC_50_ mean (± 95% CI) μM	n_(H)_ mean (± 95% CI)	Shift in potency compared to its WT-control
WT-1	− 40	4	1.49 (1.27–1.74)	0.81 (0.68–0.93)	
WT-2	− 120	4	1.05 (0.82–1.38)	0.67 (0.57–0.77)	
WT-3	− 120	94	1.25 (0.97–1.6)	0.56 (0.48–0.67)	
T623A	− 120	94	6.97 (5.80–8.38)	0.60 (0.54–0.67)	5.58
S624A	− 40	4	2.64 (2.30–3.03)	1.12 (0.94–1.30)	1.77
V625A	− 120	4	28.9 (23.4–35.6)	0.71 (0.62–0.80)	27.52
G648A	− 120	94	11.1 (9.56–12.8)	0.67 (0.61–0.73)	8.88
Y652A	− 40	4	5.09 (4.00–6.46)	0.60 (0.53–0.68)	3.42
F656A	− 120	94	176.9 (143.9–217.3)	0.55 (0.47–0.63)	141.52
