# Supplementary material for: Molecular basis of hERG potassium channel blockade by the class Ic antiarrhythmic flecainide
Source: J Mol Cell Cardiol. 2015 Sep;86:42–53. doi: 10.1016/j.yjmcc.2015.06.021 (PMC4564290; doi:10.1016/j.yjmcc.2015.06.021)
Supplement: Supplementary file 1 — Supplementary material. [file mmc1.docx]

**Molecular determinants of hERG potassium channel blockade by the Class Ic antiarrhythmic flecainide**

**Melgari: hERG inhibition by flecainide**

*Online Supplement*

**Dario Melgari MSc^1^, Yihong Zhang PhD^1^, Aziza El Harchi PhD^1^, Christopher E. Dempsey PhD^2^, Jules C. Hancox PhD, FSB, FBPhS^1*^**

^1^ School of Physiology & Pharmacology,

Medical Sciences Building,

University Walk,

Bristol,

BS8 1TD. UK

^2^ School.of.Biochemistry,

Medical Sciences Building,

University Walk,

Bristol,

BS8 1TD, UK

^*^ Correspondence to:

Professor Jules C Hancox, School of Physiology & Pharmacology, Medical Sciences Building, University Walk, Bristol, BS8 1TD, UK. Tel +44-(0)117-3312292; Fax +44-(0)117-3312288; email: [jules.hancox@bristol.ac.uk](mailto:jules.hancox@bristol.ac.uk%7d)

**Supplementary Methods**

The fractional block of I_hERG_ “tails” by the different drug concentrations studied was determined using the equation:

$FB=1- \frac{I_{hERG-Drug}}{I_{hERG-Control}}$ (1)

Where *I_hERG-Drug_* and *I_hERG-Control_* represent the amplitude of “tail” currents in the presence or in the absence of a defined concentration of drug and the ratio between these two values represented the fraction of unblocked I_hERG_ (Figure 2Bi). Flecainide block reached a steady-state within 2-3 minutes. Therefore, correction for run-down was not required or applied.

Concentration-response relations were constructed by plotting mean fractional block of I_hERG_ tails against drug concentration, and then fitting the experimental data with a standard Hill equation which yield the half-maximal inhibitory concentration (IC_50_) and the Hill coefficient (n_H_) values:

$y=1/{(1+{10}^{˄}((Log}{IC}_{50}-x)*n_{H}))$ (2)

Where *x* is the logarithm of concentration and *y* is the fractional block of I_hERG_ at a given concentration and *IC_50_* and *n_H_* are as defined above.

The voltage dependence of activation was established by using the step protocol reported in the lower portions of panels Ai and Aii of Figure S1. The normalized tail current amplitude was plotted against the testing potential at the previous depolarizing step and the experimental data points were fitted with a Boltzmann function of the form:

$I={I_{MAX}}/{{(1+exp(V_{0.5}-x}/{Slope))}}$ (3)

Where *I* is the tail current elicited after the testing voltage *x*, *I_MAX_* is the maximal current recorded, *V_0.5_* is the half activation voltage and *Slope* is the curve slope factor. The same equation was used to simulate the I-V curves showed in panel C of Figure S1.

The voltage dependence of inactivation was studied using the three step protocol showed in the inset of Figure 3A. The I_hERG_ transients at the beginning of the third step were analysed as previously described [1;2]. The normalized current was plotted against the testing voltage during the 2ms second step; experimental data were then fitted with the following Boltzmann equation:

$I/{I_{MAX}=1-(1+\exp\left[ ({V_{0.5}-V_{m})}/k \right])}$ (4)

Where *I* is the current amplitude at the beginning of the third step, *I_MAX_* is the maximal recorded current after the 2ms step at the testing potential *V_m_*, *V_0.5_* is the half-maximal inactivation potential and *k* is the slope factor for the relationship.

To obtain the time-course of inactivation at +40mV, the decay of the resurgent current at the beginning of the third step after a 2ms repolarisation step to -120mV was fitted with a single exponential equation of the form:

$y=A\times exp({-x}/{\tau)+C}$ (5)

Where *y* is I_hERG_ recorded at time *x*, *τ* is the time constant of the decay of the transient current, *A* represent the total fitted current and *C* is the residual unfitted current after the decline of the resurgent current. A similar single exponential function was used to fit the recovery from flecainide block during a long depolarizing step to 0 and +40mV.

*Docking of flecainide to a hERG homology model*

A homology model of the open configuration of the hERG channel pore tetramer (pore helix, selectivity filter and S6 helix) based on the crystal structure of the MthK channel (PDB 1LNQ; [3]) was utilized to perform docking simulations of flecainide, using methods previously conducted in our laboratory [4;5]. Drug docking in this model has been shown to broadly accord with experimental measures for a variety of hERG blockers (5;6). The structure of flecainide was built using the Biopolymer module of Insight II (Accelrys, San Diego, CA, USA), protonated on the piperidine nitrogen atom, and then exported to Sybyl-x 2.0 (Tripos, St. Louis, MO, USA) in order to calculate the partial charges using the Gasteiger-Hückel module, and to energy minimize the molecule. Docking runs with GOLD [7] were performed with sampling of side chain serine and threonine side chain hydroxyl rotational flexibility and rotamer sampling of Tyr652 and Phe656 as described in [5]. A series of docking runs was also performed using the Flexidock module of Sybyl-x 2.0 using the methods described in [5]; this analysis is described in the Supplementary results (Figure S3).

*Flecainide and flecainide analogues*

Flecainide acetate (Sigma-Aldrich) and Flecainide analogues (QX-Flec and NU-Flec [8], synthesized by Ascent Scientific) were dissolved respectively in Milli-Q water and DMSO to produce 1 and 10mM stock solutions. Stock solutions were diluted in Standard or High K^+^ Tyrode at room temperature to obtain final concentrations reported in the “Results” section. All the solutions were pre-heated to 37°C and applied to the cell under study using a homemade, multi-barrelled superfusion system which enabled a rapid (<1 s) exchange of superfusate [9].

**Supplemental Results**

Although I_hERG_ inhibition by flecainide has been reported to exhibit some voltage-dependence [10], we wanted to quantify this under the conditions of the present study. Figure S1Ai and Aii contain representative currents at selected voltages during the protocol shown under the traces (test commands were applied in 10 mV increments between -40 and +60 mV). Tail currents were normalized (see Methods) and plotted as shown in Figure S1B, then fitted with equation 3 to derive voltage-dependent activation parameters for I_hERG_. The control V_0.5_ was -18.7±2.3mV and *k* was 5.35±0.18, whilst in the presence of 1µM flecainide solid line, V0.5=-25.5±2.6mV, Slope=5.00±0.30 (n=5). Thus, flecainide induced a ~-6.5 mV left-shift in voltage-dependent activation. The V_0.5_ and *k* values derived from equation 3 were used to produce continuous plots of voltage-dependent activation in the absence and presence of drug and these were plotted together with voltage-dependence of fractional block of the I_hERG_ tail in Figure S1C. The region of voltage-dependence of I_hERG_ tail inhibition coincided closely with the rising phase of the I_hERG_ activation plot, consistent with gating (activation)-dependent block. Voltage dependence of block was consistent with, but more marked than that reported previously [10], as there was an apparent negative fractional block at ‑40 and -30mV. This resembles that seen with other hERG channel blocking agents that produce leftward shifted voltage-dependent activation (eg [11;12]), and is likely a consequence of the leftward shift in I_hERG_ activation at this voltage in the presence of flecainide.


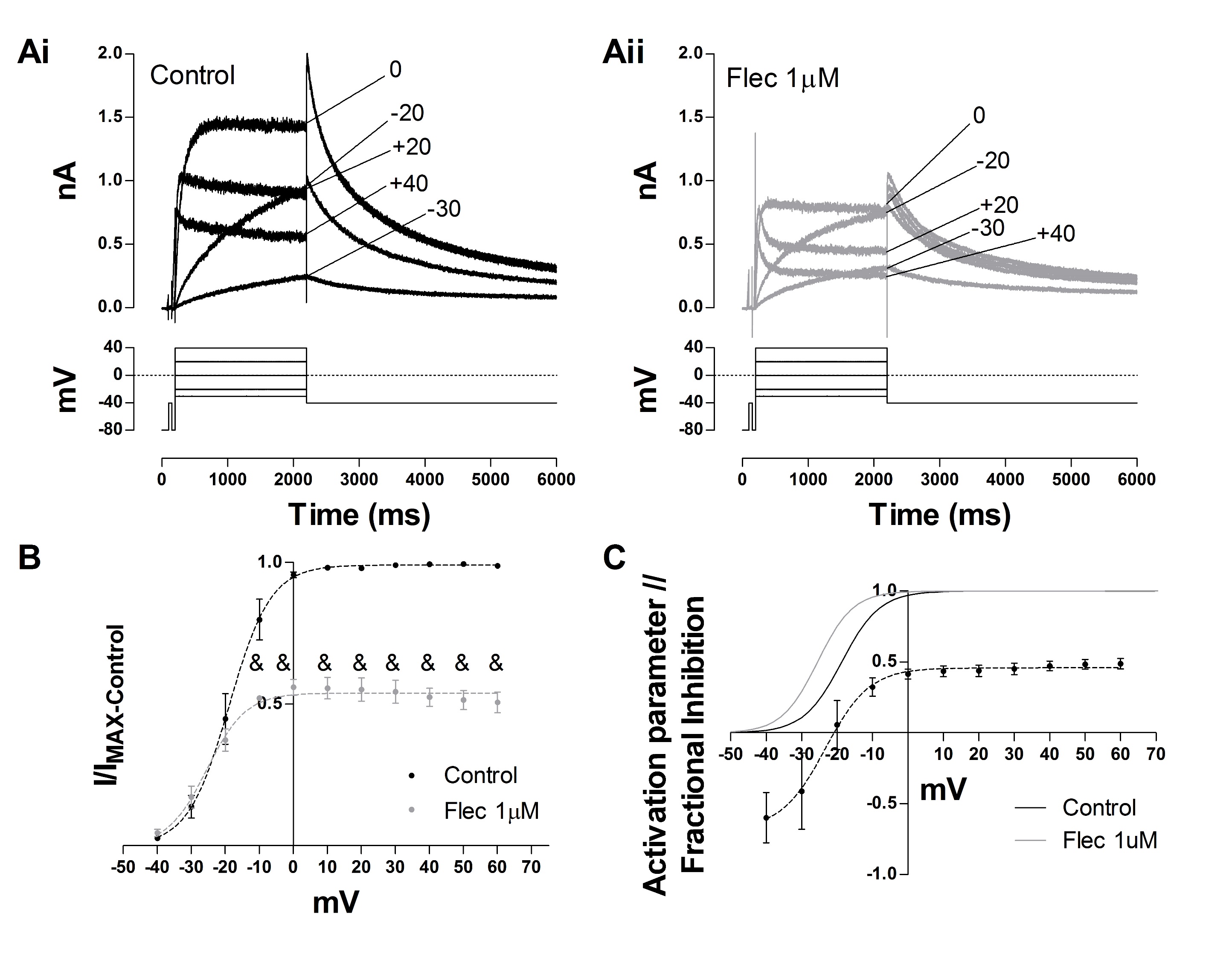


**FIGURE S1**

**A** Representative current traces of I_hERG_ elicited by the step protocol showed below in Control (**Ai**) and in the presence of 1µM flecainide (**Aii**; for clarification selected currents at 4 test voltages are shown while the full protocol spanned from -40 to +60mV with a 10mV increase at each step). Peak I_hERG_ tails at -40mV following each depolarising command were measured relative to that elicited by the initial brief 50ms step from -80mV to -40mV.

**B** Normalised I-V relationship for I_hERG_ tails in Control (black) and in presence of 1µM flecainide (grey). Peak tail currents in both conditions were normalized to the maximal tail current amplitude recorded in Control (n=5, **&** p<0.01 2-Way ANOVA with Bonferroni post hoc test). The experimental points were fitted with equation 3. Control V_0.5_ = -18.7±2.3mV, Slope 5.35±0.18 and flecainide 1µM V_0.5_ = -25.5±2.6mV, Slope 5.00±0.30. ΔV_0.5_=-6.83mV, (p<0.01 paired t-test) (n=5).

**C** Voltage dependence of flecainide block (black dotted line) and simulated voltage-dependent activation relations for I_hERG_ in Control (black line) and in the presence of 1µM flecainide. The activation relations were simulated by calculating activation variables at 2 mV intervals using equation 3 and the activation parameters yielded by experimental data.

The data with flecainide analogues (Figure 2, main manuscript) on wild-type (WT) hERG, considered together with the data on S6 alanine mutants were strongly suggestive that flecainide accesses the channel pore in its charged form, from the cell interior, and then interacts with aromatic side chains of F656 residues of the S6 domains of the channel subunits. We reasoned that if this is the case, then the F656A mutant should be relatively resistant to block by internally applied QX-Flec. Similar to the experiment on the WT channel, it was not possible for a cell to act as its own control as the cell interior became exposed to drug from the patch-pipette on gaining whole-cell access. Consequently, experiments were performed in which the magnitude (density) of inward I_hERG_ tails carried by the F656A channel were compared between groups of cells dialysed with normal patch solution (Control) and cells dialysed with QX-Flec containing solution. The results of these experiments are shown in Figure S2. There was no significant difference between current density in the two groups of cells (p>0.05, Mann-Whitney U test), indicating that QX-Flec was ineffective at inhibiting F656A hERG. In separate experiments the same experimental voltage protocol was applied to untransfected cells, with little or no current elicited, thereby confirming that the measured inward current in Control and drug from F656A hERG expressing cells was I_hERG_ with little or no contamination from overlapping endogenous current.


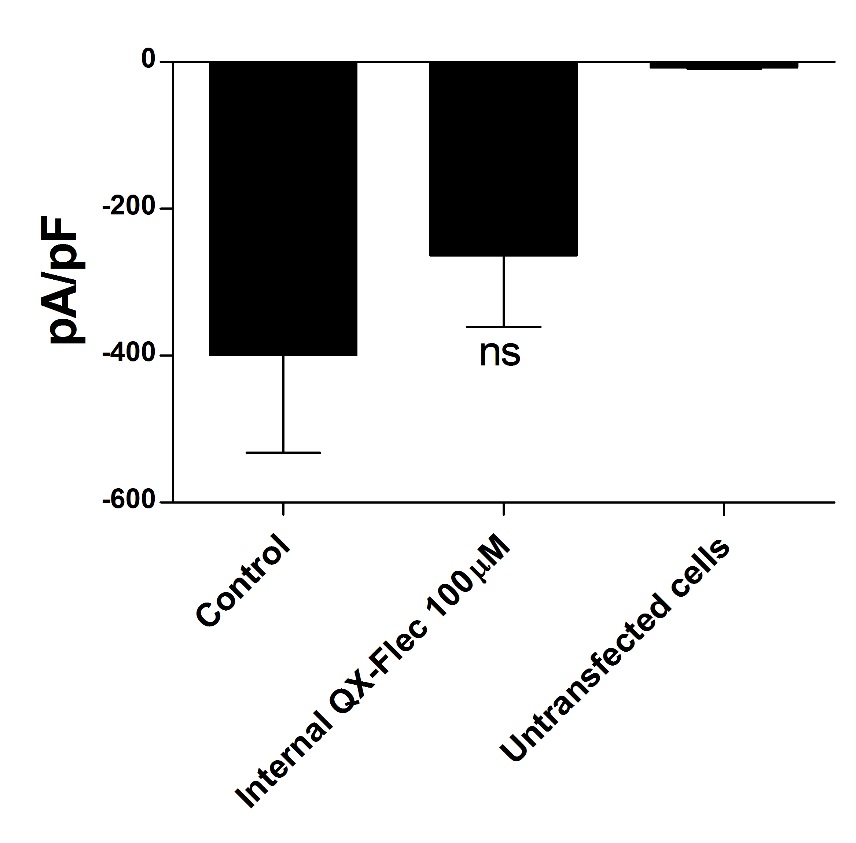


**FIGURE S2**

The effect of the F656A mutation on internally applied QX-Flec. The left and middle bars show unpaired data for the density of inward F656A I_hERG_ tails at -120 mV in the absence of any drug exposure (“Control”; n=12) and when QX-Flec was applied via the patch-pipette (“Internal QX-Flec 100 µM”; n=8). There was no significant difference in current density between the two conditions (p>0.05; Mann-Withney test). The rightmost bar shows comparable recording in the absence of drug from untransfected cells to highlight the lack of inward current with this protocol in the absence of hERG (n=9).

Canonical hERG pore blockers are usually characterized by a positively charged aliphatic ammonium that contributes an electrostatic component to binding [5;13]. Therefore, in addition to GOLD docking simulation (Figure 6), we performed a second series of docking experiments using Flexidock which incorporates an explicit parameterisation of electrostatic contributions to binding [5]. Side chain flexibility of residues T623, S624, V625, Y652, F656 and S660 was sampled during docking simulations. The genetic algorithm was run for 60,000 generations until energy score convergence was reached. Flexidock outputs are sensitive to the starting structure of the drug within the pore. Therefore, different starting positions and orientations of flecainide in the pore cavity were prepared and the docking simulation was repeated for a total of 240 runs. Flexidock ranked the docking outputs according to their energy scores and the six best low energy poses were selected for further analysis with Pymol (Schrödinger). A representative low energy pose is showed in Figure S3. Flexidock tends to dock the positively charged ammonium of hERG blocking drugs in or near the binding site for K^+^ ions (i.e. closer to the selectivity filter) [5]. However, our experiments yielded low energy poses with flecainide docked lower into the cavity and closer to the inner mouth interacting with the side chains on Y652 and F656 (Figure S3). Gold and Flexidock are not expected necessarily to yield similar outputs [5], but in this case the docking results obtained with the two programs showed consistency with one another, supporting the *in vitro* mutagenesis and electrophysiology results.

**Figure S3 Flexidock docking simulation. (A)** Lateral **(Ai)** and intracellular views **(Aii)** of a representative low energy pose of flecainide (yellow sticks) docked to an MthK-based open-state hERG channel homology model. Pore region domains are shown as grey ribbons. Residues T623, S624 and V625 are shown as green sticks, Y652 and F656 are shown as pink and blue sticks respectively. Purple spheres represent K^+^ ions in the [1] and [3] positions of the selectivity filter. **(B)** Representative Flexidock energy pose of flecainide (yellow sticks) docked to hERG pore cavity residues. Residues V625, Y652 and F656 are shown as green, pink and blue sticks, respectively. For clarity one F656 side chain which does not interact with the drug is hidden. The interactions yielded by this simulation included a π-π interaction 3.1Å between the aromatic ring of the benzamide moiety of flecainide and F656, a second π-π interaction 3.3Å between the benzamide moiety and a second F656 (black dotted line); non-polar 3.0Å interaction between flecainide CF_3_ and the aromatic ring of a third F656 (light blue dotted line).

**Table S1. Inactivation-dependence of I_hERG_ inhibition by Class I and III antiarrhythmic drugs.**

| Drug | Class | S631A | N588K | N588K/S631A | Source |
| --- | --- | --- | --- | --- | --- |
| Flecainide | Ic | 5.0 | 4.4 | 12.9 | This study |
| Quinidine | Ia | 3.5 | 3.5/5.8/1.2 | 6.6 | [14]/[15]/[16] |
| Disopyramide | Ia | 1.6 | 1.5 | 7.1 | [14] |
| Propafenone | Ic | 3.8 | 2.4 | 8.7 | [14] |
| Amiodarone | III | 4.1 | 4.2 | 29.1 | [14] |
| E-4031 | III | 12.0 | 11.4 | 36.4 | [14] |
| D-Sotalol | III | - | 20 | - | [15] |
| Dofetilide | III | 168/10 | 7.4 | - | [16-18] |

The table compares the effect of the S631A, N588K and S631A/N588K attenuated inactivation mutations on I_hERG_ block by the listed Class I and III antiarrhythmic agents. Data are shows as fold-change in IC*_50_* values compared to WT I_hERG_ (the IC_50_ mutant/ IC_50_ WT ratio). The data on flecainide come from this study. Data in [14-16] were all derived from mammalian expression systems. Data in [17] and [18] were derived from *Xenopus* oocytes**.**

**References**

[1] McPate MJ, Duncan RS, Milnes JT, Witchel HJ, Hancox JC. The N588K-HERG K^+^ channel mutation in the 'short QT syndrome': mechanism of gain-in-function determined at 37^o^C Biochem Biophys Res Comm 2005;334:441-9.

[2] DU CY, Adeniran I, Cheng H, Zhang YH, El HA, McPate MJ, et al. Acidosis Impairs the Protective Role of hERG K^+^ Channels Against Premature Stimulation. J Cardiovasc Electrophysiol 2010; 21(10):1160-9

[3] Jiang Y, Lee A, Chen J, Cadene M, Chait BT, MacKinnon R. Crystal structure and mechanism of a calcium-gated potassium channel. Nature 2002 May 30;417(6888):515-22.

[4] El Harchi A, Zhang YH, Hussein L, Dempsey CE, Hancox JC. Molecular determinants of hERG potassium channel inhibition by disopyramide. J Mol Cell Cardiol 2012; 52:185-95

[5] Dempsey CE, Wright D, Colenso CK, Sessions RB, Hancox JC. Assessing HERG pore models as templates for drug docking using published experimental constraints: the inactivated state in the context of drug block. J Chem Inf Model 2014 Feb 24;54(2):601-12.

[6] Du C, Zhang Y, El Harchi A, Dempsey CE, Hancox JC. Ranolazine inhibition of hERG potassium channels: Drug-pore interactions and reduced potency against inactivation mutants. J Mol Cell Cardiol 2014 May 27;74C:220-30.

[7] Verdonk ML, Cole JC, Hartshorn MJ, Murray CW, Taylor RD. Improved protein-ligand docking using GOLD. Proteins 2003 Sep 1;52(4):609-23.

[8] Liu H, Atkins J, Kass RS. Common molecular determinants of flecainide and lidocaine block of heart Na^+^ channels: evidence from experiments with neutral and quaternary flecainide analogues. J Gen Physiol 2003 Mar;121(3):199-214.

[9] Levi AJ, Hancox JC, Howarth FC, Croker J, Vinnicombe J. A method for making rapid changes of superfusate whilst maintaining temperature at 37^o^C. Pflugers Arch 1996;432:930-7.

[10] Paul AA, Witchel HJ, Hancox JC. Inhibition of heterolgously expressed HERG potassium channels by flecainide and comparison with quinidine, propafenone and lignocaine. Br J Pharmacol 2002;136:717-29.

[11] Ridley JM, Milnes JT, Zhang YH, Witchel HJ, Hancox JC. Inhibition of HERG K^+^ current and prolongation of the guinea-pig ventricular action potential by 4-aminopyridine. J Physiol 2003 Jun 15;549(Pt 3):667-72.

[12] Zhang YH, Cheng H, Alexeenko VA, Dempsey CE, Hancox JC. Characterization of recombinant hERG K^+^ channel inhibition by the active metabolite of amiodarone desethyl-amiodarone. J Electrocardiol 2010 Sep;43(5):440-8.

[13] Cavalli A, Poluzzi E, DePonti F, Recanatini A. Toward a pharmacophore for drugs inducing the long QT syndrome: insights from a CoMFA study of HERG K^+^ channel blockers. J Med Chem 2002;45:3844-53.

[14] McPate MJ, Duncan RS, Hancox JC, Witchel HJ. Pharmacology of the short QT syndrome N588K-hERG K+ channel mutation: differential impact on selected class I and class III antiarrhythmic drugs. Br J Pharmacol 2008;155:957-66.

[15] Wolpert C, Schimpf R, Giustetto C, Antzelevitch C, Cordeiro JM, Dumaine R, et al. Further insights into the effect of quinidine in short QT syndrome caused by a mutation in HERG. J Cardivas Electophysiol 2005;16:54-8.

[16] Perrin MJ, Kuchel PW, Campbell TJ, Vandenberg JI. Drug binding to the inactivated state is necessary but not sufficient for high-affinity binding to human ether-à-go-go-related gene channels. Mol Pharmacol 2008;74:1443-52.

[17] Lees-Miller JP, Duan Y, Teng GQ, Duff HJ. Molecular determinant of high affinity dofetilide binding to HERG1 expressed in Xenopus oocytes: involvement of S6 sites. Molecular Pharmacology 2000;57:367-74.

[18] Weerapura M, Hebert TE, Nattel S. Dofetilide block involves interactions with open and inactivated states of HERG channels. Pflugers Arch 2002;443:520-31.
